# Adolescent perception of sexual and reproductive health rights and access to reproductive health information and services in Adaklu district of the Volta Region, Ghana

**DOI:** 10.1186/s12913-023-10447-1

**Published:** 2023-12-21

**Authors:** Desmond Klu, Margaret Gyapong, Percival Delali Agordoh, Charles Azagba, Evelyn Acquah, Phidelia Doegah, Anthony Ofosu, Evelyn Korkor Ansah

**Affiliations:** 1 Institute of Health Research, University of Health and Allied Sciences, Ho, Volta Region Ghana; 2 School of Allied Health Sciences, University of Health and Allied Sciences, Ho, Volta Region Ghana; 3 Adaklu District Health Directorate, Ghana Health Service, Adaklu, Volta Region Ghana; 4 Ghana Health Service, Accra, Greater Accra Region Ghana

**Keywords:** SRH services, SRH information, Adolescent, Adaklu, Ghana

## Abstract

**Background:**

One of the key targets of Ghana’s Adolescent Health Service Policy and Strategy is to ensure that 90% of adolescents and young people have knowledge of sexual and reproductive health services and rights. This phenomenon has led to the establishment of adolescent-friendly health facilities to increase access to health information and services among adolescents. Despite these efforts, access to health information and service utilisation remains low among adolescents. Our study seeks to examine adolescents’ perception of sexual and reproductive health rights (SRHR) and access to reproductive health information and services in the Adaklu district of the Volta region of Ghana.

**Methods:**

A baseline cross-sectional household survey of 221 adolescents aged 10–19 years in 30 randomly selected communities was used. A structured questionnaire was developed and administered to the respondents. A binary logistic regression analysis was used to examine the association between adolescents’ perception of adolescent sexual and reproductive health rights (ASRHR) and access to reproductive health information and services.

**Results:**

Adolescents’ perception of SRHR was poor, and this poor perception may have been reflected in a few proportions (10%) of adolescents accessing SRH information and services. Majority (91.9%) of adolescents do not use sexual and reproductive health (SRH) services in the Adaklu district. Adolescents who attained primary education (aOR = 5.99, CI: 1.16–30.95), those who never had sexual communication with their father (aOR = 8.89, CI: 1.99–39.60) and adolescents who never experienced any form of sexual coercion (aOR = 11.73, CI: 1.61–85.68) had a higher likelihood of not utilising SRH services in Adaklu district. Regarding access to SRH information, adolescents who ever discussed sexual matters with their fathers, those who ever used contraceptives and adolescents who ever experienced sexual coercion had lower odds of accessing information on contraception, sexually transmitted infections, and teenage pregnancy.

**Conclusions:**

Access to and use of sexual and reproductive information and health services among adolescents in Adaklu district remain very low, which has implications for adolescents’ knowledge and perception of their SRHR. Considering the factors predicting this phenomenon, it is recommended that interventions can be tailored to address the unique challenges faced by adolescent in accessing comprehensive SRH support.

**Supplementary Information:**

The online version contains supplementary material available at 10.1186/s12913-023-10447-1.

## Background

Globally, the adolescent population, aged 10 to 19 years, comprises approximately 1.2 billion people, accounting for 18% of the world’s total population. Notably, nearly 90% of these adolescents reside in lower- and middle-income countries [1]. The transitional phase of adolescence is marked by significant sexual and reproductive health (SRH) challenges, which hold the potential to negatively influence the social, physical and cognitive maturation of adolescents [2, 3]. Thus, addressing these challenges remains paramount, given their pivotal role in shaping the development and advancement of adolescents. Adolescents’ health, well-being and development can be promoted through access to adolescents’ health information services [4]. Studies have underscored that the health needs, behaviours and expectations of adolescent are unique and that routine health care services are inadequately equipped to provide these services [4–6].

The Sustainable Development Goals (SDGs) and Universal Health Coverage (UHC) agendas present crucial opportunities for the realization of sexual and reproductive health rights, encompassing diverse and vulnerable populations. These agendas also provided a platform to redefine and augment the equitable access and high-quality provision of Sexual and Reproductive Health (SRH) services. According to the Convention on the Rights of the Child, persons under age 19 possess the right to health-related information and services, facilitating their survival and optimal growth to their potential. In the context of sub-Saharan Africa, adolescents are faced with a myriad of SRH challenges, such as constrained and unequal access to information and utilisation of services pertaining to unsafe abortion, sexuality, gender-based violence and family planning. A comprehensive study conducted by Guttmacher Institute revealed that as of 2019, about 41% of adolescents were confronted by numerous unmet needs for SRH services with specific emphasis on contraception [7].

The International Conference on Population and Development (ICPD), which was held in Cairo in 1993, acknowledged the adverse consequences of engaging in risky sexual behaviour by adolescents. As a response, various nations, including Ghana, were urged to implement measures aimed at addressing this phenomenon [8]. Consequently, the Government of Ghana undertook commitments and became a signatory to multiple national and international initiatives that uphold the right to sexual and reproductive health. These initiatives affirmed the entitlement of adolescents and young persons to accessible, appropriate, and high- quality healthcare facilities and services. Subsequently, Ghana took steps to establish specialized adolescent-friendly reproductive health ‘corners’ to address the needs of adolescents. These ‘corners’ have been introduced with the aim of enhancing adolescents’ access to comprehensive health information and services, reflecting the nation’s commitment to promoting the well-being of its adolescent population.

According to a report in 2015 from the Family Health Division of Ghana Health Service, there has been a limited improvement in adolescents’ access to appropriate health information over time. Although the overall utilisation of healthcare services has steadily progressed in the general population, adolescents’ uptake of freely available reproductive health information remains low [9]. The report identified several key factors contributing to these challenges. These encompass unfavorable attitudes exhibited by healthcare providers, inadequately equipped, and poorly resourced adolescent friendly health facilities, financial constraints, and a dearth of parental support. These factors collectively influence adolescents’ perceptions of health services and impede their utilisation. Consequently, these barriers to the utilisation of SRH services among adolescents have repercussions for their sexual health and well-being. Such hinderances lead to engagement in risky sexual behaviours, including induced abortions, multiple sexual partnerships, participation in unprotected sexual activity and susceptibility to Human immunodeficiency virus (HIV/AIDS) and other sexually transmitted infections (STIs).

Other studies found significant barriers to adolescents’ access to SRH information and utilisation of SRH services. These studies underscore substantial service costs, the remote location of health facilities, inconvenient service placements, inaccessible confidential sexual and reproductive (SRH) services and fear of being ridiculed as major barriers [10–14]. These supply-related factors exert a detrimental impact on the healthcare system’s capacity to provide adolescents with high-quality SRH services, thereby hindering their ability to make informed decisions about their sexual health. Addressing these challenges is pivotal to enhancing the provision of comprehensive SRH services to adolescents and ensuring their empowerment in making informed sexual health choices.

Adolescents, particularly girls residing in rural parts of developing countries, are confronted by a heightened susceptibility to reproductive health problems, including STIs, unintended teenage pregnancies, unsafe abortion practices, multiple sexual partnerships, as well as instances of sexual harassment and coercion [15]. Despite these risks, the utilisation of SRH services among adolescents remains notably low [15, 16]. Within the context of the study area, a significant proportion of female adolescents (23.3%) experienced teenage pregnancies, juxtaposed with a meager utilisation rate (10%) of SRH services [17]. In line with this situation, Ghana’s Adolescent Health and Development Program (ADHD) discerns and endeavors to address the diverse needs of adolescents. It includes the right to sexual health, fostering self-esteem, and cultivating their capacity to shoulder responsibility for themselves, their relationships, and their role in society. However, the landscape is marked by a pronounced prevalence of misconceptions and misinformation concerning matters of sexuality, fertility, and contraception in Ghana. Moreover, adolescents and young people often lack awareness of their sexual rights and the array of services accessible to them. This predicament is further exacerbated by the scarcity of knowledge and information regarding adolescents’ perceptions of their SRH rights and how these notions translate into the actual acquisition of SRH information and utilisation of services. This knowledge gap is particularly salient in rural settings, where adolescents are especially vulnerable to exploitative sexual encounters and negative sexual outcomes, such as teenage pregnancy, HIV AIDS and other STIs.

Against this backdrop, this study seeks to bridge the existing knowledge gap and contribute to literature by examining adolescents’ perception of reproductive health rights and its influence on their access to SRH information and utilisation of SRH services in the Adaklu district of the Volta region, Ghana. Our study seeks to contribute substantively to the existing body of knowledge, thereby enriching our understanding of adolescents’ reproductive health behaviours and their utilisation of vital SRH resources.

## Methods

### Study area and period

This study was carried out in Adaklu district, Volta region of Ghana, in November 2019. The district consists of 91 communities. Based on the 2010 population and housing census and according to the regional population, the district has a projected population of approximately 44,942 made up of 21,022 males and 22,920 females representing 49.0% and 51.0%, respectively. The estimated adolescent population (10–19 years) in the district is 9,887. The age-dependency ratio in the district is 72.1, the total fertility rate of the district is 2.4, and the crude birth rate is 18.3. The total number of households (HH) in the district was 35,960. The district has 6,089 households with an average of 1.1 household per house. The average household size is 5.9 persons per house, higher than the regional average of 4.3. The proportion of male household population who are heads of household (24.1%) is higher than the proportion of female household population who are household heads (10.1%) in the district [18].

The district has five health subdistricts and 15 Community-based Health Planning Services (CHPS) zones with five health centres and 11 CHPS compounds [19].

### Study design

This is a cross-sectional study, done as baseline for an adolescent reproductive health intervention project in Adaklu district of the Volta Region of Ghana. Thus, the study was drawn from a larger study on adolescent sexual and reproductive health (ASRH) issues and the perspective of their parents on ASHR.

### Study population

The study respondents were adolescents 10–19 years from 30 selected communities in the district who lived in the area for a minimum of 6 months.

### Sampling technique and sample size

The study encompassed a total of 30 communities, each with a minimum population of 500 residents in the district. Employing a simple random approach, these communities (clusters) were selected as study sites. The methodology adopted for the cluster selection was based on a modified version of the Expanded Program of Immunisation (EPI) cluster sampling technique. Within each selected community, a set of seven households were randomly selected to participate in the study. In each community we randomly selected the starter household and follow the EPI Sampling Technique to identify subsequent households. Only one adolescent per household was interviewed. The selection of the adolescents was done through the following process, the centre of the selected community was located. Then a bottle was spun at the centre of the community. The direction the bottle pointed to was identified and the fifth house in that direction selected as the starting point. A household with an eligible adolescent in the fifth house was then identified. There was a ballot to randomly select one adolescent when multiple eligible adolescents are living in the household. Instances when there are no eligible adolescents in the fifth house, the field officers come out of that house and went to the nearest house whose entrance faced them directly. Once they are able to select an eligible adolescent from the starting point, they move to the fifth house in that direction until it is exhausted. The field officers, then move to the fifth house in the opposite direction of where the spun bottle pointed to for the continued selection of the respondents. The same procedure was used to select more respondents from the other two directions of the spun bottle. Questionnaires were administered to adolescents aged 10–19 years residing in these households. In order to ensure a fair representation of both genders, a deliberate and conscious effort was undertaken to include an equal number of male and female respondents. However, in instances where a community exclusively comprised female adolescents in the selected households, a directive was issued to the field officers. This instruction prompted the selection of solely male adolescents from households in the next community. This strategy was adopted to mitigate any potential disparities in the representation of male and female adolescents, ultimately ensuring a more even-handed assessment of the subject matter under study. Similarly, participants for the baseline study were drawn from two categories: adolescents with membership in adolescent health clubs and those non-members of the clubs. The determination of the sample size was calculated using the single population proportion formula, guided by the following parameters: an anticipated proportion of 17.6%, a confidence level set at 95%, with an α (alpha) value of 0.05, a margin of error of 3% and a design effect of 2. To account for potential non-response, an additional 5% was incorporated into the initial calculation, resulting in the final sample size of 221 (7 × 30 + 11) adolescents aged 10–19 years.

### Data collection tools and procedure

A structured questionnaire was developed and administered to the respondents. The development of this questionnaire was guided by insights garnered from prior studies on adolescent sexual and reproductive health [20–22].

The main points included in the questionnaire were sociodemographic characteristics, sexual and reproductive health knowledge and services, sexuality, sexual communication between adolescents and their parents, risky sexual behavior, sexual harassment, and coercion (see Supplementary file). Data collection was conducted by seven trained research assistants recruited based on their skills and proficiency in the English and Ewe language and a supervisor for 21 days.

The questionnaire and informed consent documents were initially developed in English and later translated into the Ewe language (a widely spoken language in the communities). The questionnaire was pre-tested, after which all the necessary corrections, refinement and modifications were diligently incorporated to ensure its robustness and appropriateness.

## Measurements

### Outcome variables

The study’s outcome variables were the utilisation of SRH services and access to SRH information on contraception, STIs and pregnancy. The measurement of these outcome variables relied on the following questions:

1. *Have you ever visited a health facility or health worker of any kind to receive services on contraception, pregnancy, abortion or sexually transmitted infections?*
2. *Did any health worker or nurse talk to you about?*
  - *Contraception?*
  - *Sexually transmitted infections?*
  - *Pregnancy?*

Respondents provided either a ‘yes’ or ‘no’ response to these questions. An index was developed to represent the data, where a respondent was assigned a value of 1 for ‘yes’ if they affirmed ever accessing one or more of the aforementioned SRH services or information, and a value of 0 for ‘no’ if they responded in the negative. The measurement of the outcome variables in this study is analogous to the measurement employed in an earlier study conducted by Abajobir and Seme [23], which assessed the level of reproductive health knowledge and service utilisation among adolescents. This conformity in measurement underscores the continuity of measurement across related studies.

### Predictor variables

In this study, the main predictor of interest was the perception of Adolescent Sexual and Reproductive Health Right (ASRHR). Perceiving such rights is a complex concept to measure, as it involves a multifaceted process of selecting, organizing and interpreting information through awareness of cues and behavioural intentions that occur in social context [24]. Previous studies have examined various dimension of social perception [25, 26]. However, our study focused on specific facets, namely of opinions on dating (sexual partnership) among boys and girls, physical interaction like kissing and touching (sexual exploration) between boys and girls and adolescents using contraceptives during sexual activity (contraceptive use). These indicators are essential elements of SRHR [27].

To operationalize the measurement of adolescents’ perception of SRHR, we computed three distinct variables: the right to engage in sexual partnership, the right to sexual exploration, and the right to use contraceptives during sexual encounters. Each of these variables were measured using the following three statements: “I believe it is a right for unmarried boys and girls to have dates” (right to sexual patnership), “I believe it is a right for boys and girls to kiss, hug and touch each other” (right to sexual exploration) and “It is good for boys and girls to have sex with each other provided that they use contraceptives to stop pregnancy” (right to use contraceptives). Respondents could provide either ‘agree’ (a score of ‘1’’) or ‘disagree’(a score of “0”) responses to these statements.

The composite variable “adolescent level of perception of SRHR”was derived from these three statements. A score range of ‘3–4’ (a respondent who agreed to 2 or more of the three statements) was categorised as indicating a favourable level of preception, whereas a score range of ‘0–2’ (a respondent who disgreed with 2 or more of the three statements) was designated as indicative of a less favorable perception. This composite variable was then employed as the main predictor variable in the subsequent analyses.

Other predictor variables included in the study were background characteristics of adolescents, namely sex (male/female), age (10–14 years/15–19 years), educational level attained (no education/primary/secondary), current school attendance (yes/no), subdistricts (Sorfa-Torda/Helekpe/Ahunda/Waya), adolescent club membership (Member/Nonmember), sexual communication with father and mother (Never/Ever), ever use of contraceptives (ever use/never use), engagement in transactional sex (i.e. receive or pay money/gifts for sex) (Don’t engage/Engage) and sexual coercion which was determined by whether the respondent had experienced forceful penetrative sex or rape (Ever coerced/Never coerced), harassment which is measured by whether the adolescent had experienced touching of genitals, creasing of breast or kissing without consent (Sexually harassed/Not harassed) and intercourse experiences (ever had sex/never had sex).

### Data processing and analysis

Data collected was systematically coded and entered into the REDCap. Data quality was ensured through the application of legal values, range checks and validation rules. Data were cleaned for internal consistency and then exported to SPSS version 25 for analysis. Frequency distributions and inferential statistics were estimated using design-based analysis in the form of percentages and cross-tabulations. Bivariate analysis was employed to examine the association between the outcome and predictor variables, quantifying their statistical significance. In a binary logistic regression analysis, we conducted two different types of analysis (two models were fitted for each analysis). In the first regression analysis, we developed two models to assess the influence of the predictor variables on the outcome variable. Model I analysed the effect of adolescent perception of SRHR on access to SRH information while model II analysed the influence of adolescent perception of SRHR on access to SRH information, while controlling for the potential effect of other socio-demographic factors. In the second regression analysis, the first model examined the influence of adolescent perception of SRHR on their utilization of SRH services. On the other hand, the second model examined the combined effect of adolescent perception of SRHR and other socio-demographic factors on utilization of SRH services. For all the models, we presented adjusted odds ratios (aORs) and their associated 95% confidence intervals (CIs).

## Results

### Descriptive

#### Adolescent perception level of sexual and reproductive health rights

The findings, as presented in Table 1, revealed that a higher proportion of adolescents (55.2%) reported less favourable perception of their sexual and reproductive health rights.

**Table 1:** Background characteristics and perception level of SRHR of adolescents (10–19 years)

**Variables**	**Number of adolescents**	**Percent**
**Adolescent’s level of SRHR perception**		
More favourable perception	99	44.8
Less favourable perception	122	55.2
**Sex**		
Male	89	40.3
Female	132	59.7
**Current Age**		
10–14	100	45.2
15–19	121	54.8
**Educational Level**		
No Education	32	14.5
Primary	96	43.4
Secondary +	93	42.1
**Currently Schooling**		
Yes, full time	199	90.0
No	22	10.0
**Adaklu Subdistricts**		
Sofa Torda	44	19.9
Helekpe	106	48.0
Ahunda	35	15.8
Waya	36	16.3
**Adolescent Club Membership**		
Not a member	77	34.8
Member	144	65.2
**Sexual Communication with Mother**		
Never	143	64.7
Ever	78	35.3
**Sexual Communication with Father**		
Never	188	85.1
Ever	33	14.9
**Ever use of Contraceptives**		
Never use	179	81.0
Ever use	42	19.0
**Transactional sex (Receive money or gifts for sex)**		
Do not Engage	205	92.8
Engages	16	7.2
**Transactional sex (Pay money for sex)**		
Do not Engage	210	95.0
Engages	11	5.0
**Sexual Coercion**		
Never Coerced	205	92.8
Ever Coerced	16	7.2
**Sexual Harassment**		
Not sexually Harassed	176	79.6
Sexually Harassed	45	20.4
**Ever had Sexual Intercourse**		
Never had sex	172	77.8
Ever had sex	49	22.2

Source: 2019 IDRC Adolescent Health Intervention Project

#### Sociodemographic characteristics

In terms of the respondents’ background characteristics, the results showed that 59.7% of the respondents were girls, with the remaining 40.3% being boys. Almost equal proportions of adolescents had completed primary (43.4%) and secondary (42.1%) levels of education, while the least proportion comprised adolescents without any formal education (14.5%). Similarly, majority (90%) of these adolescents were attending school.

A higher proportion (48%) of sampled adolescents resided in the Helekpe subdistrict, which stands out as the most urbanised subdistrict in the study area. It was observed that almost two-thirds (65.2%) of adolescents aged 10–19 years are members of adolescents’ health clubs operating in the district. Moreover, approximately 65% and 85% of adolescents reported not engaging in conversations with their mothers and fathers, respectively, regarding matters related to sexuality. Contraceptive use was observed to be low among adolescents in the district, with 8 out of 10 adolescents confirming that they have never used any form of contraception. Notably, majority of adolescents do not engage in sexual activities for transactional purposes. In addition, the occurrence of sexual harassment and coercion is relatively low among adolescents in the district, with approximately 80% and 90% of adolescents having never experienced such instances. Engagement in sexual intercourse was relatively low among adolescents, with 22.2% reporting that they have ever been involved in sexual activities.

#### Adolescents’ perception of their sexual and reproductive health rights

**Fig. 1:**
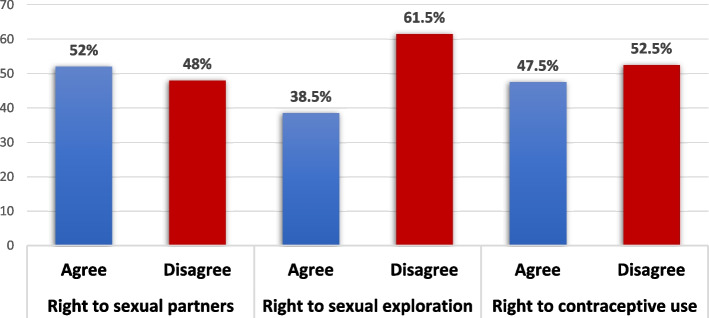
Perception of adolescents on their sexual and reproductive health rights. Source: 2019 IDRC Adolescent Health Intervention Project

Figure 1 illustrates the’ perception of each of the three ASRHRs. These are the right to have sexual partners, the right to sexual exploration and the right to use contraceptives. More than half (52%) of adolescents ‘agreed’ that they have the right to have sexual partners, and 38.5% and 47.5% also agreed that they have rights to sexual exploration and contraceptive use, respectively.

#### Adolescent utilisation of sexual and reproductive health services and types

**Fig. 2:**
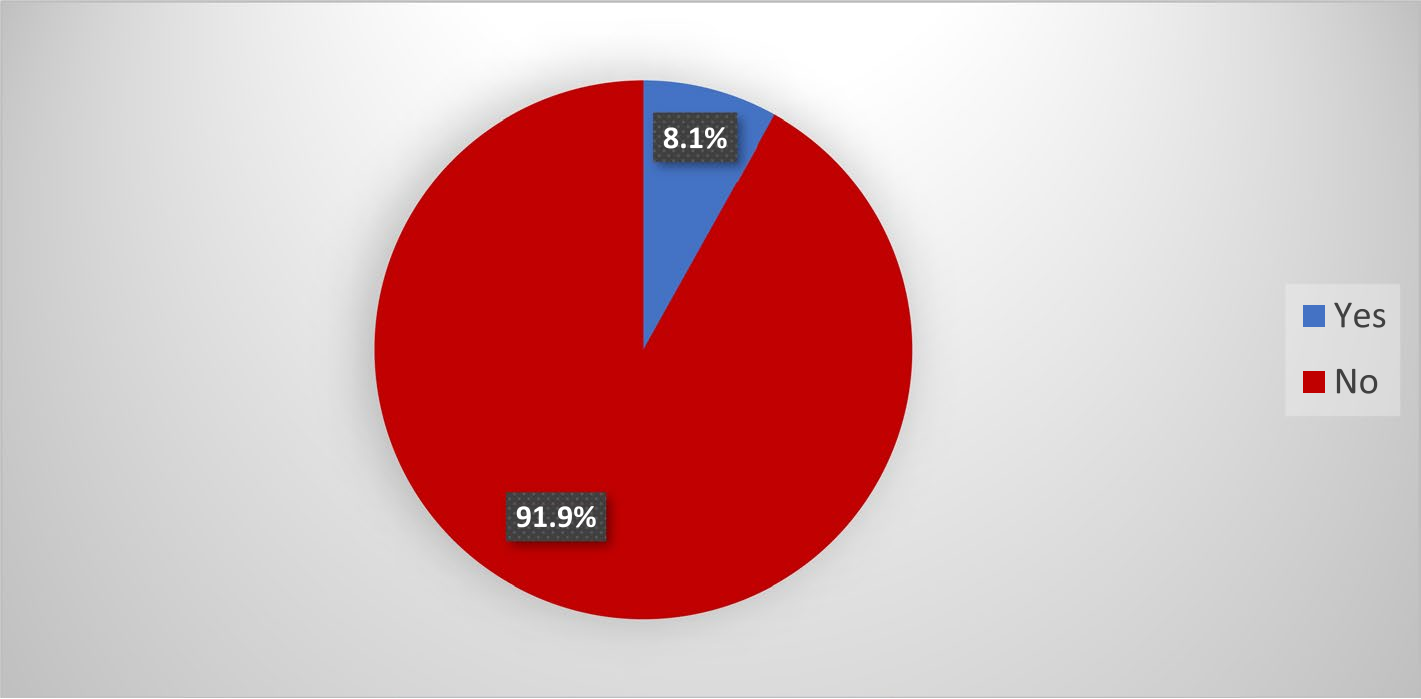
Adolescent utilisation of sexual and reproductive healthcare services. Source: 2019 IDRC Adolescent Health Intervention Project

**Fig. 3:**
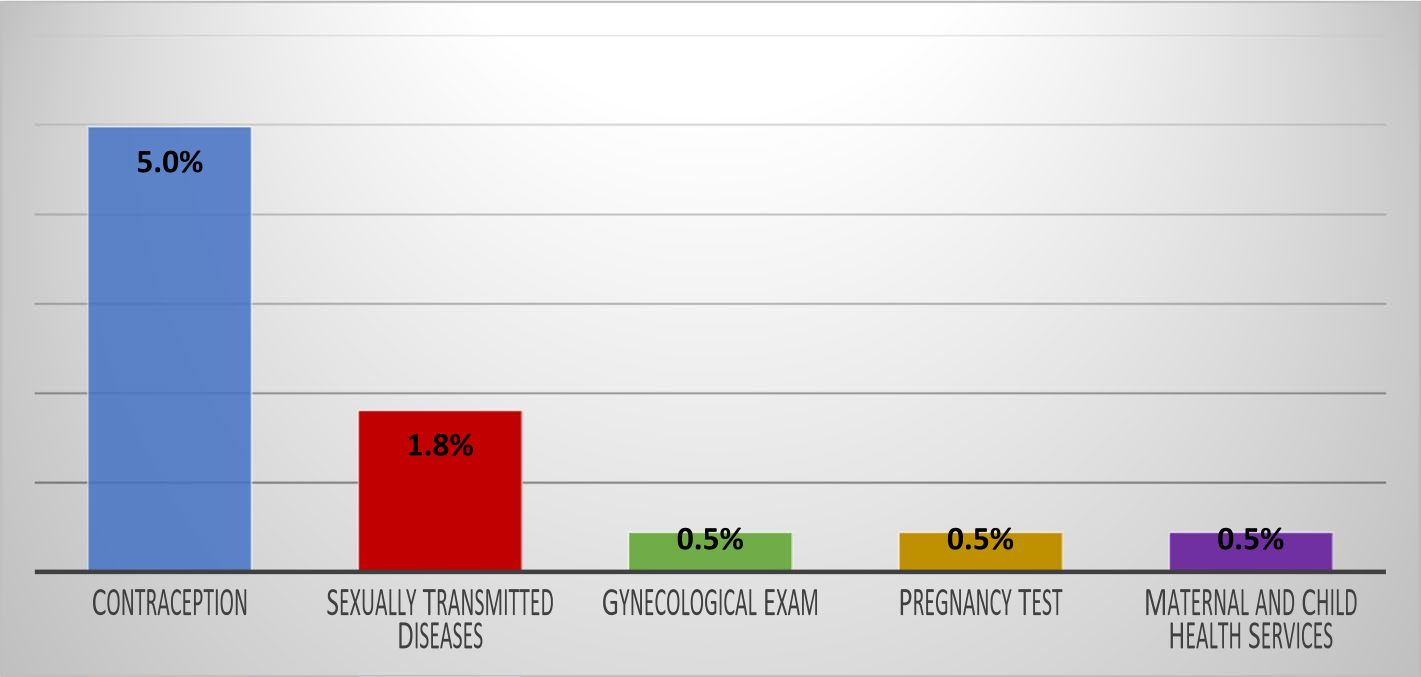
Types of sexual and reproductive health services. Source: 2019 IDRC Adolescent Health Intervention Project

Figures 2 and 3 depicts the proportion of adolescents using SRH services and the types of services mostly used respectively. Only 8% of adolescents reported that they have used SRH services. Out of the proportion utilising these health services, 5% sought services on contraception, 1.8% on sexually transmitted infections and less than 1% (0.5%) of adolescents reported going to health facilities for gynaecological exams, pregnancy tests and other maternal and child health services, as depicted in Fig. 3.

#### Association between level of perception of ASRHR, background characteristics of adolescents (10–19 years) and utilisation of sexual and reproductive health services

Age, educational level, current schooling status, living in the subdistrict, sexual communication with father and mother, ever use of contraceptives, engagement in transactional sex (pay or receive money/gifts), sexual harassment and sexual intercourse showed statistically significant associations (*P* < 0.05) with low SRH service utilisation as shown in Table 2.

**Table 2:** Adolescent (10–19) utilisation of sexual and reproductive health services according to their level of SRHR perception and background characteristics

**Characteristics**	**Utilisation of Sexual and Reproductive Health Services**				
	**Yes**		**No**		
	**%**	**n**	**%**	**n**	***p*** ** value (X** ^**2**^ **)**
**SRHR perception level**					
More favourable	13.1	13	86.9	86	0.155
Less favourable	7.4	9	92.6	113	
**Sex**					
Male	5.6	5	94.4	84	0.077
Female	12.9	17	87.1	115	
**Current Age**					
10–14	4.0	4	96.0	96	**0.007**
15–19	14.9	18	85.1	103	
**Educational Level**					
No Education	9.4	3	90.6	29	**0.023**
Primary	4.2	4	95.8	92	
Secondary +	16.1	15	83.9	78	
**Currently School**					
Schooling	8.0	16	92.0	183	**0.004**
Not Schooling	27.3	6	72.7	16	
**Adaklu Subdistricts**					
Sofa Torda	6.8	3	93.2	41	**0.042**
Helekpe	11.3	12	88.7	94	
Ahunda	0.0	0	100.0	35	
Waya	19.4	7	80.6	29	
**Adolescent Club Membership**					
Not a member	7.8	6	92.2	71	0.432
Member	11.1	16	88.9	128	
**Sexual Communication with Mother**					
Never	7.0	10	93.0	133	**0.046**
Ever	15.4	12	84.6	66	
**Sexual Communication with Father**					
Never	6.9	13	93.1	175	**0.000**
Ever	27.3	9	72.7	24	
**Ever use of Contraceptives**					
Never used	4.5	8	95.5	171	**0.000**
Ever use	33.3	14	66.7	28	
**Transactional sex (Receive money or gifts for sex)**					
Do not Engage	7.8	16	92.2	189	**0.000**
Engages	37.5	6	62.5	10	
**Transactional sex (Pay money for sex)**					
Do not Engage	9.0	19	91.0	191	**0.049**
Engages	27.3	3	72.7	8	
**Sexual Coercion**					
Never Coerced	7.3	15	92.7	190	**0.000**
Ever Coerced	43.8	7	56.3	9	
**Sexual Harassment**					
Not sexually Harassed	6.3	11	93.8	165	**0.000**
Sexually Harassed	24.4	11	75.6	34	
**Ever had Sexual Intercourse**					
Never had sex	4.1	7	95.9	165	**0.000**
Ever had sex	30.6	15	69.4	34	

Source: 2019 IDRC Adolescent Health Intervention Project

The low utilisation of SRH services was higher among adolescents aged 10–14 years while high proportion of adolescents with no formal education (90.6%) and primary education (95.8%) recorded low use of SRH service. Similarly, in-school adolescents (92.0%) had the highest proportion of low SRH service use. Adolescents who indicated never communicating with their mother (93.0%) and father (93.1%) on sexual issues recorded a high rate of low utilisation of SRH services as shown in Table 2.

Regarding the ever use of contraception, the proportion of low use of SRH was high among adolescents who never used contraceptives (95.5%). Again, the prevalence of low SRH utilisation is high among adolescents who indicated they have not engaged in any form of transactional sex (92.2%) (pay or receive money/gifts in exchange for sex). A higher proportion of respondents who did not experience any form of sexual coercion (92.7%) and harassment (93.8%) recorded a low SRH service utilisation rate. Similarly, low SRH service utilisation was high among adolescents who had never had sexual intercourse (95.9%).

#### Factors influencing the low utilisation of sexual and reproductive health service among adolescents aged 10–19 in Adaklu district

Table 3 presents two models of a binary logistic regression analyses of factors that predict the likelihood of low sexual and reproductive health (SRH) service utilisation among adolescents in Adaklu district. The first model regressed the effect of only the ASRHR perception level on SRH service uptake, and the second model included adolescents’ background characteristics together with the ASRHR perception level. In the first model, the ASRHR perception level was not significant in predicting low SRH service among adolescents.

**Table 3:** Multivariate regression analysis showing ASRHR perception level and other factors associated with utilisation of sexual and reproductive health services among adolescents aged 10–19

**Factors**	**Odds ratio**	**95% confidence interval**
***Model I***		
**ASRHR perception level**		
More favourable	0.53	0.22–1.29
Less favourable *(Ref)*	1.00	
***Model II***		
**ASRHR perception level**		
More favourable	1.01	0.30–3.42
Less favourable *(Ref)*	1.00	
**Sex**		
Male	0.60	0. 15–2.48
Female *(Ref)*	1.00	
**Age**		
10–14	1.47	0.31–7.12
15–19 *(Ref)*	1.00	
**Educational Level**		
No Education	0.99	0.14–6.99
Primary	5.99*	1.16–30.95
Secondary + *(Ref)*	1.00	
**Currently Schooling**		
Schooling *(Ref)*	1.00	
Not Schooling	0.29	0.06–1.36
**Adolescent Club Membership**		
Not a Member	1.83	0.42–8.03
Member *(Ref)*	1.00	
**Sex Communication with Mother**		
Never	1.13	0.29–4.47
Ever *(Ref)*	1.00	
**Sex Communication with Father**		
Never	8.89**	1.99–39.60
Ever *(Ref)*	1.00	
**Ever Use Contraceptives**		
Never used	8.16	0.42–160.41
Ever use *(Ref)*	1.00	
**Transactional Sex (Receive money or gifts for sex)**		
Do not Engage	1.96	0.32–12.08
Engage *(Ref)*	1.00	
**Transactional Sex (Pay money for sex)**		
Do not Engage	1.23	0.16–9.64
Engage *(Ref)*	1.00	
**Sexual Coercion**		
Never Coerced	11.73*	1.61–85.68
Ever Coerced *(Ref)*	1.00	
**Sexual Harassment**		
Not Sexually Harassed	1.42	0.31–6.41
Sexually Harassed *(Ref)*	1.00	
**Ever had Sex Intercourse**		
Never	0.51	0.02–12.50
Ever *(Ref)*	1.00	

Source: 2019 IDRC Adolescent Health Intervention Project

^*^ *P* < 0.01

^**^ *P* < 0.05

^***^ *P* < 0.001

In the second model, the likelihood of low SRH service uptake was approximately 6 times higher among adolescents who attained a primary level of education (aOR = 5.99, 95% C. I: 1.16–30.95) relative to adolescents who attained secondary or higher education. The odds of low SRH service utilisation were approximately 9 times higher among adolescents who never discussed sexual issues with their father (aOR = 8.89, 95% C. I: 1.99–39.60) compared with where there was adolescent-father communication on sexual issues.

The likelihood of low SRH service utilisation was also significantly predicted by adolescents who had never experienced any form of sexual coercion (aOR = 11.73, 95% CI: 1.61–85.68) compared to those who were victims of sexual coercion.

#### Factors affecting access to information on contraception, sexually transmitted infections and pregnancy among adolescents aged 10–19 years in Adaklu district

The multivariate results for access to information on contraception, sexually transmitted infections and pregnancy in Table 4 reiterate that some relevant factors, such as adolescents’ sexual communication with their fathers, ever use of contraceptives and sexual coercion, were found to be significant predictors of SRH information access among adolescents.

**Table 4:** Multivariate regression indicating factors affecting access to information on contraception, sexually transmitted disease and pregnancy among adolescents aged 10–19

**Factors**	**SRH info on Contraception**		**SRH info on Sexually Transmitted Disease**		**SRH info on Pregnancy**	
	**Odd ratios**	**95% CI**	**Odd ratios**	**95% CI**	**Odd ratios**	**95% CI**
**ASRHR perception level**	*Model I*					
More favourable	0.69	0.24–1.98	1.09	0.36–3.25	0.63	0.24–1.65
Less favourable *(Ref)*	1.00		1.00		1.00	
	*Model II*					
**ASRHR perception level**						
More favourable	1.68	0.39–7.17	2.22	0.57–8.62	1.48	0.41–5.31
Less favourable *(Ref)*	1.00		1.00		1.00	
**Sex**						
Male *(Ref)*	1.00		1.00		1.00	
Female	3.02	0.52–17.50	2.80	0.52–15.00	2.23	0.47–10.69
**Age**						
10–14 *(Ref)*	1.00		1.00		1.00	
15–19	0.68	1.00–4.71	0.59	0.09–3.99	0.31	0.05–2.04
**Educational Level**						
No Education	1.08	0.04–2.23	2.16	0.17–27.61	0.77	0.89–6.66
Primary	6.92**	1.04–46.23	9.28**	1.13–76.43	3.39	0.66–17.42
Secondary + *(Ref)*	1.00		1.00		1.00	
**Currently Schooling**						
Schooling	2.02	0.29–14.65	1.70	0.27–10.68	1.76	0.31–9.94
Not Schooling *(Ref)*	1.00			1.00	1.00	
**Adolescent Club Membership**						
Not a member *(Ref)*	1.00		1.00		1.00	
Member	0.45	0.07–2.82	0.72	0.15–3.58	0.39	0.08–1.96
**Sexual Communication with mother**						
Never *(Ref)*	1.00		1.00		1.00	
Ever	3.12	0.50–19.51	1.26	0.25–6.29	1.75	0.38–8.11
**Sexual Communication with father**						
Never *(Ref)*	1.00		1.00		1.00	
Ever	0.04***	0.01–0.24	0.11**	0.02–0.61	0.09***	0.02–0.48
**Ever use Contraceptives**						
Never use *(Ref)*	1.00		1.00		1.00	
Ever use	0.13**	0.03–0.65	0.18**	0.04–0.91	0.14**	0.03–0.59
**Transactional sex (Receive money or gifts for sex**						
Do not engage *(Ref)*	1.00		1.00		1.00	
Engage	0.60	0.06–6.27	0.62	0.07–5.91	0.56	0.08–3.76
**Transactional sex (Pay money for sex)**						
Do not engage *(Ref)*	1.00		1.00		1.00	
Engage	0.47	0.05–4.57	0.48	0.05–4.51	0.57	0.08–4.35
**Sexual Coercion**						
Never Coerced *(Ref)*	1.00		1.00		1.00	
Ever Coerced	0.09**	0.01–1.02	0.12**	0.01–1.04	0.12*	0.02–0.95
**Sexual Harassment**						
Not sexually Harassed *(Ref)*	1.00		1.00		1.00	
Sexually Harassed	2.24	0.29–17.57	1.94	0.26–14.53	1.25	0.23–6.71

Source: 2019 IDRC Adolescent Health Intervention Project

^*^ *P* < 0.01

^**^ *P* < 0.05

^***^ *P* < 0.001

#### Predictors of access to information on contraception among adolescents aged 10–19 years

From the results, adolescents with a primary educational level (aOR 6.92, 95% CI: 1.04–46.23) were more likely to have access to information on contraception. Adolescents who had ever discussed sexual issues with their father (aOR = 0.04, 95% CI: 0.01–0.24) were less likely to receive information on contraceptives than those who never had sexual communication with their fathers. Adolescents who ever used contraceptives (aOR = 0.13, 95% CI: 0.03–0.65) and ever experienced sexual coercion (aOR = 0.09, 95% CI: 0.01–1.02) were less likely to access information on contraception in health facilities.

#### Predictors of access to information on sexually transmitted infections (STIs) among adolescents aged 10–19 years

The probability of accessing STIs information in health facilities was higher (aOR = 9.28; 95% CI: 1.13–76.43) among adolescents with a primary level of education. Again, the odds of accessing information on sexually transmitted infections at health facilities were lower (aOR = 0.11, 95% CI: 0.02–0.61) among adolescents who never had any discussion with their fathers concerning sexual issues. Adolescents who had ever used contraceptives had a lower likelihood (aOR = 0.18; 95% CI: 0.04–0.91) of accessing information on STIs. Again, the probability of receiving STI information in health facilities was lower among adolescents who had ever been victims of sexual coercion (aOR = 0.12, 95% CI: 0.01–1.04).

#### Predictors of access to information on pregnancy among adolescents aged 10–19 years

Accessing information on pregnancy at health facilities had a lower probability among adolescents who had ever discussed sexual issues with fathers (aOR = 0.09, 95% CI: 0.02–0.48) compared to adolescents who never had a conversation with their fathers on sexual issues. Again, the odds of accessing information on pregnancy in health facilities were lower (aOR = 0.14; 95% CI: 0.03–0.59) among adolescents who had ever used contraceptives. Similarly, the odds of receiving information on pregnancy among adolescents who had ever experienced sexual coercion (aOR = 0.12, 95% CI: 0.02–0.95) were low compared to those who never sexually coerced.

## Discussion

In this study, we examine the influence of adolescents’ perception of sexual and reproductive health rights and their background characteristics on access to SRH information and utilisation of SRH services in the Adaklu district of the Volta region, Ghana. Our findings underscored a prevalent deficiency in adolescents’ perception of their sexual and reproductive health rights. A significant proportion of adolescent expressed disagreement with regards to various rights, such as their right to choose sexual partners, their right to contraceptive use, and their right to sexual exploration. This concerning to lack of recognition of these rights could lead to all forms of negative outcomes, including sexual violence, adverse health consequences and reduced engagement with SRH services [13]. Similar findings were recorded in previous studies [23–25], where factors such harmful traditional practices, lack of privacy, perception of high severity of SRH issues, and unprofessional conduct by service providers were identified as key reasons for the low utilisation of SRH service among adolescents. Additionally, other studies highlighted challenges such as inadequately equipped adolescent health facilities, distant health facilities and the high cost of services [11, 13] as significant barriers to SRH service utilisation. Coupled with earlier findings [13, 24, 25], our study, emphasizes the existence of a considerable unmet need for SRH services among adolescents particularly in the district, Ghana.

With regards to predictors of SRH services utilisation among adolescents, the study revealed that adolescents who attained primary level of education were more likely to utilise SRH services compared to those with secondary education. These findings are consistent with previous studies [27–30], which also identified educational level as a significant predictor of SRH service utilisation among adolescents. These studies found that higher educational levels were associated with a greater likelihood of utilising SRH services. This pattern contradicts the typical assumption that higher education correlates with greater SRH service utilisation. The possible explanation offered for this outcome is that adolescents with primary education are more likely to be in the 10–14 age group where they are still learning the basic rudiments of adolescence and exploring their sexual and reproductive health. This basic knowledge could make them more aware of the importance of accessing such services. Furthermore, it is suggested that adolescents who complete primary education possess a fundamental understanding of SRH concept but might lack the comprehensive knowledge needed to make fully informed decisions. On the other hand, those with secondary education tend to perceive themselves to have more understanding or control of their sexuality but this contributes to them taking more sexual risky behaviours.

Our study also found that adolescents who never communicated with their father on sexual issues had higher probability of utilising SRH services compared to those who had communicated with their fathers on sexual issues. Contrary findings were reported in a study conducted by Bhatta and colleagues [31] in Nepal which indicated that adolescent who often communicate with their parents (fathers) were more likely to use SRH services. Other studies did not find any significant relationship between parent-adolescent communication on sexual issues and utilisation of adolescent-friendly SRH services [32, 33]. An array of plausible explanations can be given to elucidate the findings of this study. Adolescents who never communicated with their fathers about sexual issues may lack accurate information about SRH. This lack of information could lead to a higher likelihood of engaging in risky sexual behaviour resulting in unintended pregnancies or the need for SRH services.

The study further showed that adolescents who were not coerced into sexual activities had higher odds of utilising SRH services compared to those had ever experienced sexual coercion. These findings are in line with previous research studies [34–36] which observed lower utilisation of SRH services among adolescents and young people who were victims of sexual exploitation. The study suggests that one possible explanation for this pattern is that adolescents who have experienced sexual coercion might associate sexual activities with negative emotions such as fear, shame and guilt. These negative emotions could potentially discourage them from seeking SRH services, as they might be hesitant to confront the emotional baggage tied to their past experiences. Furthermore, adolescents who have not experienced sexual coercion may have healthier self-esteem and a stronger sense of agency and control over their bodies and decisions. This psychological resilience and autonomy could lead them to be more willing to seek SRH services when needed, as they are accustomed to making independent choices that prioritise their well-being.

Concerning adolescents’ access to SRH information on contraception, STIs and pregnancy, educational level of the adolescent was significant in predicting this outcome. The odds of adolescents with primary level of education accessing information on contraception and STIs is higher compared to adolescents with secondary education level. These findings align with similar results were reported by other studies [28–30], which also observed that adolescent s with lower education levels were more likely to seek information from alternative sources. One possible explanation for this phenomenon is that adolescent with lower levels of education often come from disadvantaged socio-economic backgrounds. They may have limited access to formal education and resources, which could lead them to rely more heavily on community health programs and other sources of information on contraception, pregnancy and STIs.

This study found that sexual communication with fathers, ever use of contraceptives and experiences of sexual coercion significantly predicted adolescents’ likelihood of accessing information on contraception, teenage pregnancy and STIs in health facilities. Specifically, adolescents who had ever discussed sexual matters with their fathers had lower odds of visiting health centres for information on these topics. These findings are consistent with previous studies [37–39], where adolescents who received SRH information from their parents tended to perceive that there was no need to visit health centres for such information. One possible explanation for this outcome is that adolescents who experience open communication about sexual matters with their fathers may feel more comfortable and informed about these topics due to the guidance they receive at home. As a result, they might perceive less urgency in seeking additional SRH information from health facilities. This underlines the important role that parental communication plays in shaping adolescents’ attitudes and behaviours regarding sexual health. Additionally, within the context of the study area, the overwhelming respect for fathers and ehat they represent in the lives of growing adolescents is enough deterrent against socially reprehensible behaviour. This is especially so when it is the father who initiates such a discussion. The discourse may typically include an exposition on the father’s expectation of the child, an agreed reward for achieving the recommended “good behaviour” as well as unambiguous severe sanctions, including freeze of financial support and even getting disowned, for walking the lane of socially undesirable behaviour. It is imperative to note that the socialization in patriarchal cultures like the one in the Adaklu district place significant socio-cultural premium on identity- even more so in being publicly disowned by one’s biological father.

Another important finding of this study is that adolescents who have ever used contraceptives are less likely to visit health facilities for information on pregnancy, STIs and contraception. This finding contrasts with the findings of other studies [40], that indicated a positive relationship between health facility visits and contraceptive use. This implies that visits to health facilities for information on these topics can positively impact contraceptive utilisation among adolescents. In other words, receiving comprehensive SRH information from health facilities could potentially lead to more informed choices and increased contraceptive use among adolescents.

Additionally, the study found that adolescents who had ever experienced sexual coercion were less likely to receive SRH information at health facilities. This is a significant observation, as experiences of sexual coercion can have profound psychological and emotional impacts on adolescents. Such experiences might lead to normalised attitudes about abusive sexual encounters, misinformation among about SRH, feelings of shame, and many misconceptions about sex [3, 41]. This, in turn, can hinder their willingness to seek SRH information and services from health facilities.

In areas with high sexual vulnerability, adolescents continue to engage in risky sexual behaviours despite the existence of adolescent health clubs and dedicated friendly corners for adolescents at health centres. This indicates that while these resources exist to increase access to SRH information and services for adolescents, additional factors might be influencing their decision-making and behaviour. Overall, the findings of this study contribute to knowledge on understanding the complex interplay of adolescents’ perception of their SRH rights and factors that affects adolescents’ access to and utilisation of SRH information and services in a high teenage pregnancy setting.

## Conclusion

In conclusion, the study conducted in the Adaklu district sheds light on a low level of awareness and perception among adolescents regarding SRHR. Despite not finding a statistically significant relationship between perception of ASRHR and utilisation of SRH information and services, the study recommends the design of targeted policies and educational programs aimed at educating adolescents on their SRH rights. Such efforts would serve to empower adolescents, foster personal growth, and safeguard them against exploitation, deception or mistreatment. The study also revealed that access to SRH information and utilisation of related services remained low among adolescents in the study area. Remarkably, about 9 out of 10 adolescents has never visited health facilities to seek SRH information and services. The most accessed SRH services by adolescents in the district included contraception, treatment for STIs, gynaecological examinations, pregnancy tests and maternal-child services. Factors such as adolescents who had engaged in open discussion about sexual matters with father (parent), ever used contraceptives, and had experienced sexual coercion were found to be predictors of low access to SRH information. Conversely, adolescents with a primary level of education, those who had not discussed sexual matters with their fathers, and those who had not experienced sexual coercion were more likely to avoid utilising SRH services. These findings are particularly relevant to similar study areas characterised by high rates of teenage pregnancy.

In the light of these findings, it is recommended that interventions can be tailored to address the unique challenges faced by adolescents in accessing comprehensive SRH support. This is will ultimately enhance adolescents’ understanding of their rights, promoting easy of communication with their parents about sexual matters and mitigating factors that hinder SRH information access and service utilisation.

### Supplementary Information

**Additional file 1.** University Of Health And Allied Sciences, Ho – Ghana. “Strengthening the capacity of Health Management Teams to use DHIMs data in engaging stakeholders for effective decision-making in addressing teenage pregnancy in the Volta region of Ghana.”

## Data Availability

Data obtained for the study are not publicly available for ethical reasons but are available upon reasonable request from the corresponding author.

## Abbreviations

AIDS: Acquired Immune-deficiency Syndrome
HIV: Human Immunodeficiency Virus
aOR: Adjusted Odds ratio
IDRC: International Development Research Centre
REC: Research Ethics Committee
SPSS: Statistical Package for Social Sciences
SRH: Sexual and Reproductive Health
STI: Sexually Transmitted Infection
UHAS: University of Health and Allied Sciences
